# Method for Assessing the Integrated Risk of Soil Pollution in Industrial and Mining Gathering Areas

**DOI:** 10.3390/ijerph121114589

**Published:** 2015-11-13

**Authors:** Yang Guan, Chaofeng Shao, Qingbao Gu, Meiting Ju, Qian Zhang

**Affiliations:** 1 College of Environmental Science and Engineering, Nankai University, Tianjin 300071, China; E-Mails: kiranedved@yeah.net (Y.G.); jumeit@nankai.edu.cn (M.J.); 2 Department of Soil Pollution and Control, Chinese Research Academy of Environmental Sciences, Beijing 100012, China; E-Mails: guqb@craes.org.cn (Q.G.); zhangqian@craes.org.cn (Q.Z.)

**Keywords:** industrial and mining gathering areas, soil environmental risk assessment, polluting factory, inherent risk level assessment, integrated risk analysis

## Abstract

Industrial and mining activities are recognized as major sources of soil pollution. This study proposes an index system for evaluating the inherent risk level of polluting factories and introduces an integrated risk assessment method based on human health risk. As a case study, the health risk, polluting factories and integrated risks were analyzed in a typical industrial and mining gathering area in China, namely, Binhai New Area. The spatial distribution of the risk level was determined using a Geographic Information System. The results confirmed the following: (1) Human health risk in the study area is moderate to extreme, with heavy metals posing the greatest threat; (2) Polluting factories pose a moderate to extreme inherent risk in the study area. Such factories are concentrated in industrial and urban areas, but are irregularly distributed and also occupy agricultural land, showing a lack of proper planning and management; (3) The integrated risks of soil are moderate to high in the study area.

## 1. Introduction

Industrial and mining activities have always been leading sources of soil pollution [1,2]. In China, mining and industrial gathering areas have been established and rapidly developed across the country. According to several studies [3,4,5] and the National Soil Pollution Survey Bulletin [6], over ten million hectares of land in China have been threatened by soil pollution. Among these, two million hectares are threatened by mining and five million are threatened by petroleum pollution. Moreover, because of irrational planning and rough development in the initial construction stage, types, range and potential risks of the pollutants in soil of industrial and mining gathering areas are intricate and should not be underestimated.

In the interests of preventing and controlling soil pollution caused by industrial and mining activities, several studies have conducted ecological and health risk assessments, evaluation criteria grading, and spatial distributions of pollutants in different regions of China. Since the 1990s, China has developed quality standards for general soil environments [7] and for soils allocated to specific land usages [8,9,10,11]. Li *et al.* [12] summarized the published data (2005–2012) on soils polluted with heavy metals originating from mining areas in China, and then comprehensively assessed the heavy metal pollution derived from these mines based on soil pollution levels and human health risks. In previous studies, the mines and surrounding areas were identified as sources and heavy metals were considered the most serious pollutants. The spatial distribution [2,13,14,15], risk assessment [2,13,16,17,18], and mobility [19,20] of pollutants in soils also provided important information.

However, the risk assessment of soil pollutants has focused almost exclusively on heavy metal pollution. Organic pollution, especially the organic pollutants generated by the petrochemical industries, is largely ignored, despite its strong presence in the industrial and mining gathering areas and its potential for serious harm. Moreover, although most researchers have analyzed the risk sources, the survey, classification, grading, and management methods of polluting factories (the main sources of pollution in industrial and mining gathering areas) are relatively simple and not associated with the risk analysis. Finally, the difficulty of investigation and data collection has precluded a spatial analysis that combines a soil pollution study with an evaluation of the inherent risk level of polluting factories. Therefore, to understand the overall status of the soil environment in industrial and mining gathering areas, a comprehensive method that considers the soil risk caused by complex pollutants and pollution sources is necessary.

The present study proposes a comprehensive method for evaluating soil risk status. It includes the human health risk, the inherent risk level of the polluting factories and evaluated risk regionalization and characteristics of pollution sources. The method was piloted in Binhai New Area, Tianjin, China, a typical area of high mining and industrial activity.

## 2. Methodology

This study assesses human health risk and the inherent risk levels of polluting factories. From these results, a comprehensive method for assessing hazardous soil environments in industrial and mining gathering areas is developed.

### 2.1. Human Health Risk Assessment

By assessing human health risk, we can characterize the potential health hazards imposed by environmental pollution and elucidate the impacts and damage to human health [21,22]. The latter (including the carcinogenic and non-carcinogenic health risks of soil contamination) are revealed by the land use patterns and exposure pathways. Appendix A describes the assessment of human health risk on both organic pollutants and heavy metal contaminants. This assessment provides a scientific basis and technical support for comprehensive risk management.

### 2.2. Inherent Risk Assessment of Polluting Factories

Polluting factories, refer to factories engaged in industrial production or other industries, which may directly or indirectly cause large-scale environmental or ecological pollution. As mentioned in the Introduction, mining and industrial activities are major sources of soil contamination in industrial and mining gathering area. The operating conditions, pollutant emission levels, environmental management, and risk prevention levels of polluting factories are all important affecters of soil environmental risks. Therefore, to guide the soil environmental management in industrial and mining gathering areas, the risk assessment of polluting factories should be included in the soil environmental risk assessment.

#### 2.2.1. Evaluation Index System

The polluting factories in industrial and mining gathering areas significantly differ in type, pollutant emission characteristics, and risk supervision level. Therefore, the environmental risks also differ among factories. The evaluation system is divided into three levels. The first layer, referred to as the target layer, measures the overall level of soil environmental risk posed by polluting factories. The second layer, the criteria layer, includes the inherent degree of sudden environmental risks, degree of cumulative environmental risk, and degree of environmental risk supervision by factories. The third level includes the assessment indicators. Appendix B described the construction of indicator system and the weight distribution of indicators, the results are listed in Table 1.

#### 2.2.2. Scoring of Indicators and Comprehensive Assessment

Because the dimensions of each index in the index system are variable, these indices cannot be directly calculated and must instead be standardized. In this study, the indicators were scored and standardized by referencing the national standards and evaluation guidelines of related industries. The standardized indicators and calculation method of parameters are presented in Appendix C.

### 2.3. Spatial Analysis

To guide the functional zoning of contaminated soil environment and identify the primary areas of soil contamination management, we require spatial analysis, risk regionalization, and a comprehensive risk partitioning method. In the current study, the human health risks were quantified by sampling, surveying, and analyzing the soil pollutants, soil environment, and the integrated status of the industrial and mining gathering areas. The assessment methods are described in Section 2.1. The results were then spatially interpolated using the inverse distance weighted (IDW) method, implemented in the ArcGis 9.3 software environment (Spatial Analyst module, ESRI, Beijing, China).

**Table 1:** Risk assessment indicators of polluting factories.

Target Layer	Criteria Layer	Indicators
Indicators of polluting factories risk assessment	Inherent level of sudden environmental risk (0.30)	Inventory level of hazardous substances (0.35)
		Service life of equipment (0.1)
		Environmental emergency response plan (0.15)
		Emergency rescue personnel (0.2)
		Environmental emergency drills frequency (0.05)
		Number of environmental emergencies in last three years (0.15)
	Level of cumulative environmental risk (0.30)	Industrial policy requirements (0.06)
		Construction Period (0.02)
		Industrial output value (0.08)
		Annual production time (0.04)
		Annual emissions of soot (0.15)
		Annual emissions of sulfur dioxide (0.15)
		Annual emissions of nitrogen oxide (0.15)
	Supervision level of environmental risk (0.40)	Industrial water recycling rate (0.05)
		Utilization rate of industrial solid waste (0.05)
		Online sewage monitoring system (0.15)
		Routine environmental monitoring capacity (0.15)
		Rain and sewage system (0.05)
		Ground seepage treatment (0.1)
		Treatment rate of soot (0.15)
		Treatment rate of sulfur dioxide (0.15)
		Treatment rate of nitrogen oxide (0.15)

Compared with the IDW interpolation, other commonly employed methods such as Kriging and Spline interpolation also have strong ability to predict the overall trend of soil pollution. However, in purpose of identifying of the polluted areas, it is necessary to require the interpolation method to predict the local feature of soil pollution. In industrial and mining gathering areas, the concentration of pollutants in soil showed a high spatial variability, but the local maxima of soil pollution (concentration or risk value) is likely to be smoothed out by Kriging or Spline interpolation. Therefore, to reserve the local maxima and minima of soil pollution in industrial and mining gathering areas, IDW interpolation is an appropriate choice. Moreover, relevant study [23] indicated that, according to the root mean square error (RMSE) for cross validation, although Kriging and Spline interpolation are more accurate than other methods, the interpolation results of soils in polluted area estimated by Kriging are significantly smaller than the results by actual statistical results. Therefore, as a measure of overall sample prediction accuracy, RMSE cannot describe the estimated error of local extreme values.

From the interpolation results, the spatial distribution maps of human health risk were derived. Again, using the spatial interpolation, the assessment results of the polluting factories were embedded in an integrated risk regionalization map. In this way, the soil environment risks and inherent risks of the polluting factories were combined into a comprehensively partitioned classification of the soil environmental risks in the study area.

### 2.4. Comprehensive Analysis

The classification of land use in industrial and mining gathering areas was performed based on landscape, distribution and characteristics of population, functional requirements of lands, and protection requirements of ecological sensitive targets. Different types of land are divided into four classes: industrial land, including industrial land and mining sites, storage land, supply facilities area and so on; agriculture land, including farmland, orchards, aquaculture bases and so on; residential land, including residential areas, living areas, culture and entertainment land, education and health land, business area and so on; conservation land, including nature conservation objectives, coastal waters, wetlands and so on.

The regionalization results of human health risk (*TCR* value) and the land use class of the study area were incorporated into a matrix assessment method. This method uses the comprehensive risk classifications in Table 2 to evaluate the risk status of the industrial and mining gathering areas. From the classification results, risk management and control measures can be designated.

**Table 2:** Classification method of soil integrated risk.

Human Health Risk (*TCR*)	Land Use			
	Industrial Land	Agriculture Land	Residential Land	Conservation Land
Low risk	Low risk	Low risk	Moderate risk	High risk
Moderate risk	Low risk	Moderate risk	Moderate risk	High risk
High risk	Moderate risk	Moderate risk	High risk	Extreme risk
Extreme risk	High risk	High risk	Extreme risk	Extreme risk

### 2.5. Site Description

**Figure 1:**
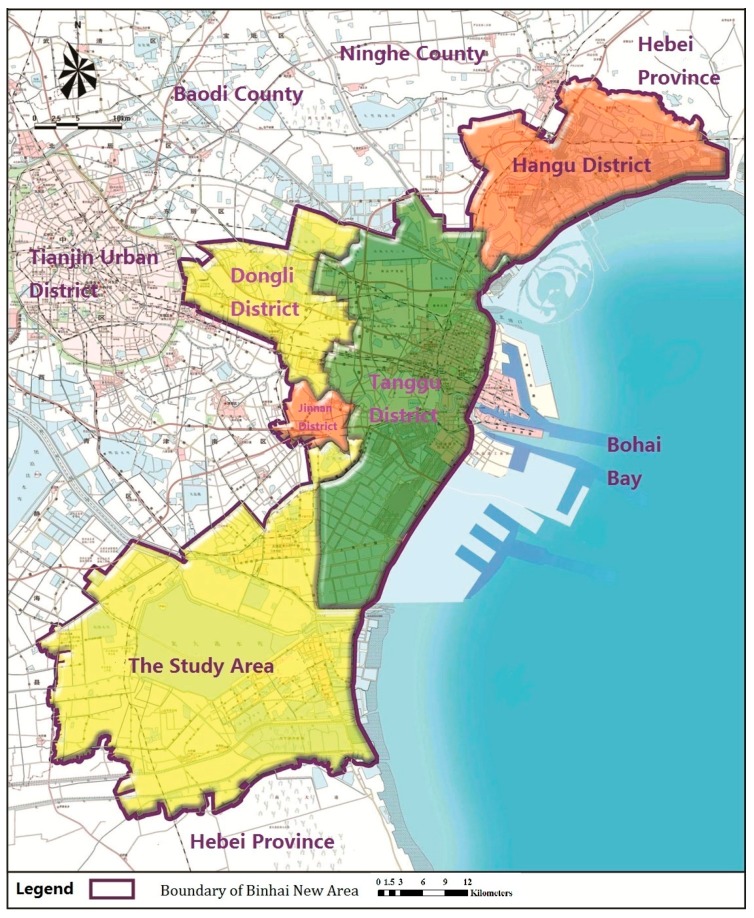
Location and scope of the study area.

The selected study area is a typical mining and industrial gathering area in the Binhai New Area, Tianjin, China, located southeast of Tianjin, China. The study area, shown in Figure 1, mainly covers the southern region of this area. Established in 1994, the Binhai New Area has become an important industrial and economic center in Tianjin, one of China’s largest industrial cities. The area is also the third zone especially designated for industrial economy development in China [24]. Unfortunately, industrial economic expansion and progress of the mining industry has been accompanied by increased soil contamination (mainly heavy metals). The study area covers approximately 1200 km^2^ and experiences a warm temperate, semihumid continental monsoon climate. Its average annual temperature and precipitation levels are 14 °C and 600 mm, respectively [25]. As noted in reports on National Major Function-oriented Zoning, China has invested heavily in developing this international port city as an eco-city and in enhancing the northern economic center of Tianjin. Owing to its rich reserves of oil and metal resources near Bohai Bay, the Binhai New Area is of significant strategic interest. The main industries are located in the northeastern and eastern parts of the region and include petrochemical, metallurgical, and mining industries. In particular, this area is becoming an important petrochemical industry base in northern China, as outlined in the Overall Plan for the New Town in Tianjin (2006–2020).

The ecological environment of this area is extremely sensitive and fragile because it borders the river, sea, and land. The pollution problem is exacerbated by the uneven distributions of the residential and industrial regions. Heavy metals introduced to the soil by human activity have contaminated large portions of this area and its vicinities [26,27,28]. Specifically, rivers, farmlands, and coastal waters have been polluted to varying extents by heavy metals discharging into water bodies over long periods.

### 2.6. Sampling and Analysis

**Figure 2:**
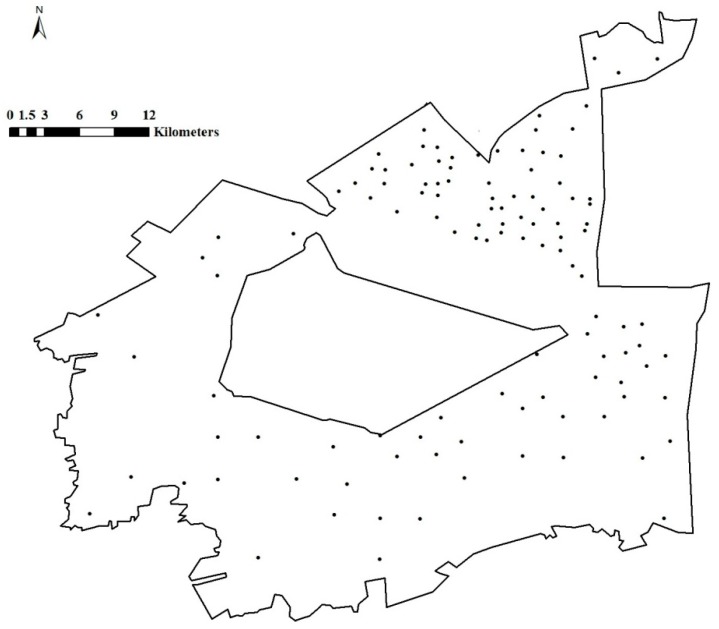
Soil sampling bitmaps. Note: the blank area is the North Dagang Reservoir.

Soil samples were collected from the study area in 2013. Forty-six census points were selected by systematic random grid sampling, the grid spacing of census points was 3 km. These points were separated by soil type, topographic characteristics, and the distribution of their contamination sources by a grid laying method. Moreover, to fully reflect the impact of highly aggregated mining industries on the quality of the soil environment, 68 encrypted points were selected in densely mined areas and areas with industrial activity (Figure 2), the grid spacing of encrypted points was 1 km. The large empty area in the sampling point map is occupied by a water reservoir.

The selected monitoring targets were eight common heavy metals, namely, As, Cd, Cr, Cu, Ni, Pb, Zn, and Hg; two common pollutants of mining industries, Co and V; and eight organic pollutants including Pyrene, Carbon tetrachloride, Dichloroethane, 1,2-, Trichlorothane, 1,1,1-, Benzene, Ethylbenzene, Fluoranthene, and Xylenes. The soil samples were pretreated by air-drying at room temperature. Plant roots, organic residues, and visible intrusions were removed from the samples. Finally, the samples were crushed, ground, and passed through a 0.85-mm sieve. The samples were suspended in deionized water (1:2.5 *v*/*v*), agitated for 1 h using a Sartorius PB-10 (Sartorius, Beijing, China), and subjected to pH measurements. The concentrations of the monitoring targets (except As and Hg) were determined by inductively coupled plasma-atomic emission spectrometry. The As and Hg concentrations were measured by atomic fluorescence spectrometry (AFS-2202, Haiguang Company, Beijing, China). Volatile and semivolatile organic compounds, polycyclic aromatic hydrocarbon substances, and other organic pollutants were monitored by gas chromatography mass spectrometry.

## 3. Results

### 3.1. Overview of Soil Pollutants

Table 3 showed the basic situation of various pollutants in soil. The detection rate of nine kinds of heavy metals reached 100%, and the detection rate of Hg and eight kinds of organic pollutants was less than 10%, indicating that heavy metal pollution was more serious in soil environment of the study area. Moreover, coefficients of variation of nine kinds of heavy metals except Hg were relative low, indicating that the nine kinds of heavy metals were widely distributed in the study area and the content difference of the nine heavy metals at different sampling points were relatively small.

**Table 3:** Overview of soil pollutants.

Item	Average (mg/kg)	Minimum (mg/kg)	Maximum (mg/kg)	Coefficient of Variation	Detection Rate
pH	8.0878	7.74	9.64	0.045598	/
As	27.5926	10	369	1.234824	100.00%
Cd	0.501	0.15	3.42	1.017565	100.00%
Cr^6+^	0.1917	0.05	0.62	0.439645	100.00%
Co	19.34	12.1	55.4	0.21456	100.00%
Cu	28.745	15.1	44.5	0.210611	100.00%
Pb	21.432	13.7	71.6	0.364035	100.00%
Ni	38.766	22.4	543	1.163055	100.00%
V	87.1395	55.7	123	0.149473	100.00%
Zn	95.233	53.3	322	0.317936	100.00%
Hg	0.0064	0	0.15	3.75	7.69%
Pyrene	0.0018	0	0.23	11.25	0.77%
Carbon tetrachloride	0.0004	0	0.05	11.00	0.77%
Dichloroethane, 1,2-	0.0012	0	0.1	8.18	1.54%
Trichlorothane, 1,1,1-	0.0084	0	0.35	0.24	5.38%
Benzene	0.0013	0	0.1	8.24	1.54%
Ethylbenzene	0.0167	0	0.77	4.74	9.23%
Fluoranthene	0.0025	0	0.32	11.27	0.77%
Xylenes	0.0888	0.03	1.39	1.77	9.17%

### 3.2. Human Health Risk

#### 3.2.1. Overview

The *TCR* and *THI* values of heavy metal contaminants and organic pollutants were separately calculated, as described in Section 2.1. The *THI* values of organic pollutants were below 1 at all sampling points, and the total *TCR* values were lower than 10^−6^. Therefore, the health hazards posed by organic pollutants in the study area were generally acceptable. The *THI* values of heavy metals were also below 1 at all sampling points, indicating that heavy metals pose acceptable non-carcinogenic risk. However, in the carcinogenic risk category, the *TCR* values of Cd, As, and Cr^6+^ at many of the sampling points exceeded 10^−6^. The results confirmed that heavy metals are the most important risk factors in the study area, especially considering their bioaccumulative character and non-biodegradability. Therefore, heavy metal pollution should continue to be targeted in industrial and mining gathering areas.

#### 3.2.2. Human Health Risk of Heavy Metals

The *TCR* ranges of Cd, Cr^6+^ and As were 1.6 × 10^−6^ to 3.8 × 10^−4^, 2.2 × 10^−8^ to 8.3 × 10^−6^, and 6.0 × 10^−6^ to 7.6 × 10^−3^, respectively, indicating that the carcinogenic risks of these three heavy metals exceeded the acceptable level by varying degrees. At 114 of the sampling points, the *TCR* of Cr^6+^ was lower than 1 × 10^−5^, indicating that Cr^6+^ poses a low carcinogenic risk. The *TCR*s of As and Cd exceeded 1 × 10^−4^ at 47 and 37 of the sampling points, respectively. Therefore, these heavy metals pose high or extreme carcinogenic risk at more than 30% of the sampling points.

**Figure 3:**
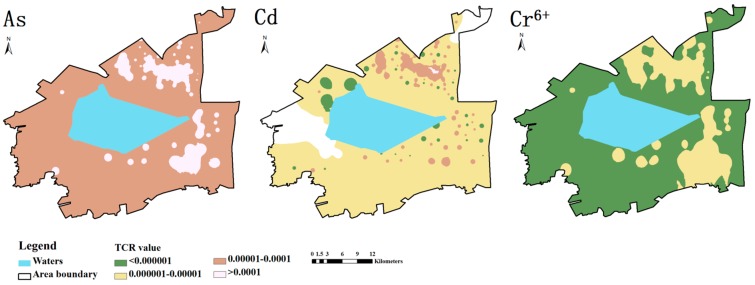
Spatial distribution map of the human health risks of three heavy metals posed by contaminated soil.

Figure 3 showed the spatial distribution of carcinogenic risks of Cd, As and Cr^6+^. It is clear that the carcinogenic risk of Cr^6+^ is low to moderate across most of the study area, the carcinogenic risk of As is high to extreme, and the carcinogenic risk of Cd is moderate to high. The overall level of carcinogenic risk of As is high, indicating that As is the main contributor of carcinogenic risk in the study area. Extreme risk areas of As are mainly concentrated in the north and east of the reservoir. Similarly, high risk areas of Cd and moderate risk areas of Cr^6+^ are mainly concentrated in the north and east of the reservoir, indicating that carcinogenic risk in such areas is more serious. Extreme risk areas of Cd are mainly concentrated in the northeast and southwest of the study area.

**Figure 4:**
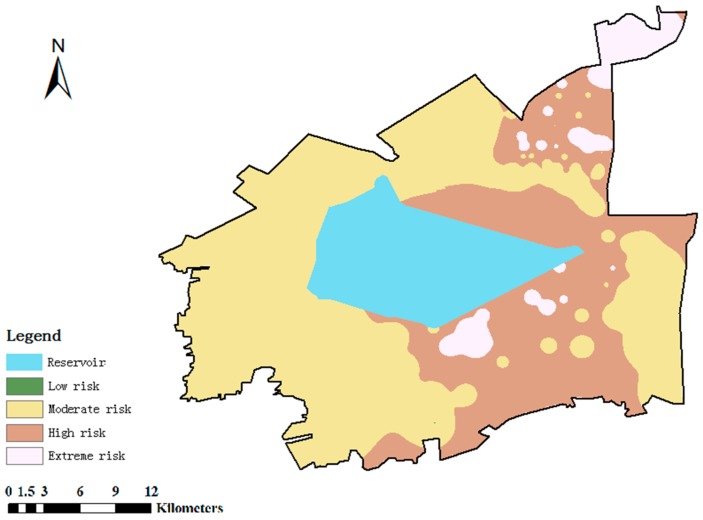
Spatial distribution map of the human health risks posed by contaminated soil.

**Figure 5:**
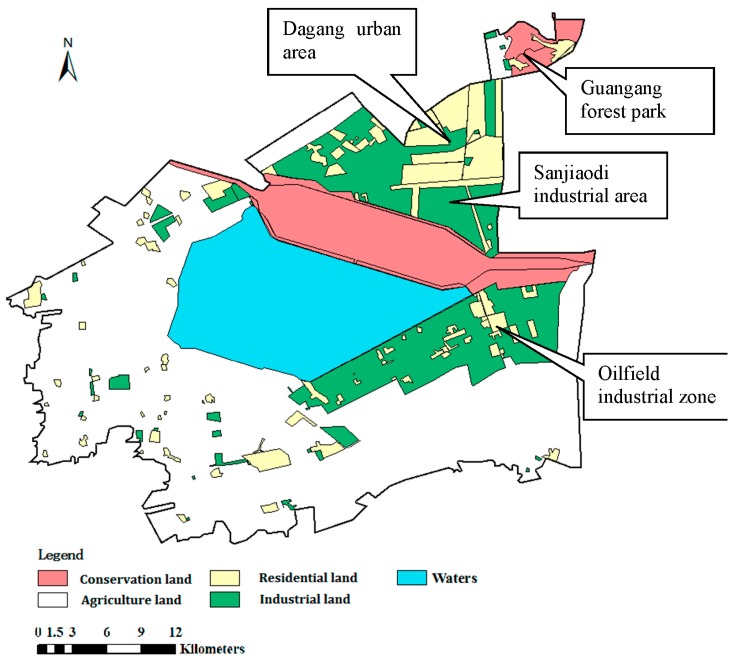
Land use of study area.

The spatial distribution of the integrated human health risk (carcinogenic), obtained by IDW interpolation of the calculated *TCR* values, is shown in Figure 4. The human health risk regionalization results revealed the following: (1) The carcinogenic risk is moderate to high risk across most of the study area; low-risk sites are minimal. (2) Extreme risk areas are mainly concentrated in the northeast of the study area and south of the reservoir. (3) Looking at the land use patterns (Figure 5), high-risk areas are found to be concentrated in the Sanjiaodi industrial area located northeast of the reservoir and in the oilfield industrial zone, with its surroundings (including residential areas). (4) Extreme risk areas are concentrated in the Dagang urban area in the northeast of the study area, the Guangang forest park in the northeast corner, and residential areas affiliated with the oilfield industrial zone. The industrial land uses of these three regions share several common characteristics; complex population composition, frequent living activities, proximity to conservation projects (reservoirs and forest parks), and high vulnerability of receptors. Risk management and pollution prevention in such areas, especially in residential lands, is essential.

### 3.3. Inherent Risk of Polluting Factories

According to the Census of Pollution Sources conducted by local environmental protection departments and statistical agencies in 2010, 150 polluting factories exist in the study area. The offending industries include electrical power, metallurgy, non-ferrous metals, petrochemicals, manufacturing (brick, paper, and textiles), and a small number of food production industries. The inherent risk of these polluting factories was calculated by the abovementioned risk evaluation method.

The calculated risks of the 150 polluting factories are summarized in Table 4. As shown in this table, none of the factories incurred no or low risk, indicating that the polluting factories pose a serious threat in the study area. Half (75) of the factories, especially those involved in smelting, forging, production and processing of non-ferrous and ferrous metals, and some petrochemical factories, posed moderate risk. These industries are the leading industries in typical industrial and mining gathering areas. However, some of the large-scale metal production and processing factories achieved low scores in environmental management system and risk prevention measures. This indicates that after 30 years of development and construction, some leading industries had gradually developed and improved their risk monitoring and prevention systems. On the other hand, a large proportion of the 150 factories were assessed as high-risk. Among the 69 factories in this category, the vast majority was brick production and thermoelectric industries; the remainder included chemical industrial factories and metal production and processing factories. Therefore, large state-owned factories remain important sources of pollution in the study area. However, note that a significant number of small-scale factories involved in property management, food production, and light industry were also high-risk. These results highlight the importance of reasonable risk control measures and risk prevention awareness. Among the six extreme risk factories, four were large state-owned petrochemical factories; the remaining two were the largest brick factory in the study area and a glass factory. The pollutant emission levels of these six factories were also high.

**Table 4:** Overview of polluting factory risk in the study area.

Polluting Factory Risk Level	Number of Factories	Main Industries
Low risk (0 ≤ *CRI* ≤ 1)	0	/
Moderate risk (1 < *CRI* ≤ 2)	75	Metal forging, processing and production; chemical industry
High risk (2 < *CRI* ≤ 2.5)	69	Brick production; thermoelectric
Extreme risk (2.5 < *CRI* ≤ 3)	6	Chemical industry

### 3.4. Integrated Risk

**Figure 6:**
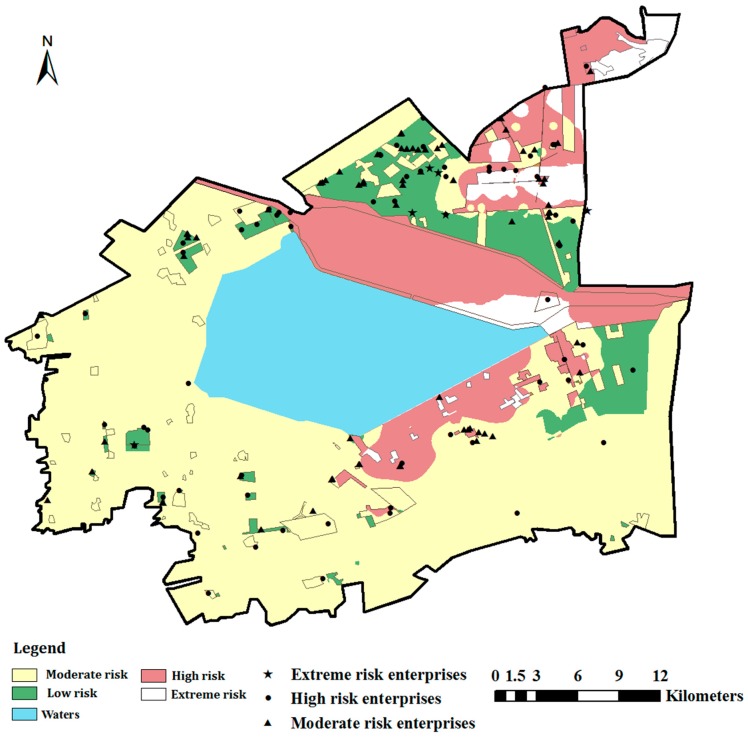
Spatial distribution map of the integrated risk.

The classification and spatial distribution of integrated risk of soil environment in the study area is presented in Figure 6. The integrated risk of soil in the study area was moderate to high. Furthermore, in Dagang urban area that located in the northeast of the study area, living areas affiliated to oilfield industrial zone that located in the southeast of the reservoir, wetland and forest park in the northeast of the study area, the integrated risk of soil was high to extreme, indicating that the integrated risks of residential land and conservation land were relative high. In most areas in the west and south of the study area, the integrated risk of soil was generally moderate. In Sanjiaodi industrial area and industrial production area of oilfield industrial zone, the integrated risk of soil was generally low.

More than half of the polluting factories were located in the northeast of the study area, and nearly 20 of these were located in residential areas, posing high to extreme threats to human health. Five of the polluting factories assessed as extreme risk were located in the Sanjiaodi industrial area; the remaining one was surrounded by several polluting factories of different sizes in the southwest of the study area. Polluting factories tended to be distributed throughout green spaces or agricultural lands in the western parts. Such occupancy of non-industrial land by factories typifies industrial and mining gathering areas throughout China, indicating unreasonable planning of land layout and a disordered distribution of factories in the initial stage of regional development. Polluting factories posing high and moderate risks were cluttered and many were located in non-industrial areas. In Figure 6, we can see that most of the polluting factories are centralized in the three industrial zones, but some are scattered outside of these zones, indicating a need for rational planning and management. Finally, although few of the polluting factories posed extreme risk to human health, their presence around densely populated areas and conservation regions such as reservoirs and forest parks presents a high inherent risk.

## 4. Discussion

The results revealed that the integrated risk status of residential lands and conservation projects were relatively high and should be improved to ensure that residents are not exposed to contaminated soil and to ensure ecological security. The rational planning of industrial and mining factories is the most important measure against soil environmental risk. In addition, polluting factories near residential lands and conservation areas should maintain their inherent risk below moderate levels. To achieve this goal, they require more comprehensive risk management plans and control measures.

The areas situated northeast to the study area, where polluting factories are largely intermingled with the population and the land use is mainly residential, were assessed as high to extreme integrated risk. Due to the high population density, such areas should be treated as the primary targets for soil risk management and control. A buffer zone should be erected between the urban and industrial areas and the emissions of polluting factories should be monitored.

Polluting factories, especially industrial and mining factories with high or extreme levels of inherent risk, should be relocated to the three major industrial areas to centralize their management and control. Industrial and mining factories located in the southwest of the study area should be shut down or relocated; alternatively, a new industrial zone should be established in this region and the residents should migrate to other residential areas. The non-negligible risks posed by other factories, including brick, food production, papermaking, and property management companies, require special attention. Such factories share common features such as cluttered distribution, lack of proper planning, and poor management. However, the production conditions and requirements and product flow of these industries largely differs from those in the heavy industrial and mining category. Therefore, relocating these factories to the three major industrial zones is neither practical nor wise. One possible solution is to establish specialized industrial areas for light industry and food production and processing in suitable locations. Moreover, these factories require guidance and supervision of their production processes, pollution monitoring systems, and environmental management measures.

From the distribution of industrial land and polluting factories in the southwest of the study area, the staggered distribution of industrial and residential lands was a prominent feature of China’s old industrial areas, and may have derived from the expansion of residential areas and functional areas of factory employees. Such intermingling reflects a lack of overall planning and long-term consideration of risk prevention. To protect residential lands, the long-term planning of industrial and mining gathering areas should separate residential and industrial lands as much as possible. Efficient transportation systems and risk isolation measures would ensure the normal operation of industrial and mining factories without posing risks to the nearby inhabitants.

## 5. Conclusions

This study established a method for assessing the soil environment risk in industrial and mining gathering areas. To this end, the pollutants and their sources were monitored and investigated. Moreover, the soil environmental risks in a typical industrial and mining gathering area were systematically analyzed. The main contributions of the study are summarized below.

(1) To assess the impacts and damage to human health by soil environmental pollution, a human health risk of heavy metal contaminants and organic pollutants was conducted. Similar to previous studies, heavy metals were identified as the most serious contaminants in the study area. High and extreme risk was found mainly in industrial and residential areas.

(2) The inherent risk level of polluting factories, which pose the main risks in industrial and mining gathering areas, was evaluated. The evaluation system was designed to optimize the layout of the regional environmental risk sources while protecting the residential population and the most sensitive conservation targets.

(3) A comprehensive analysis of soil environmental risk was conducted using a matrix overlay. By this method, the integrated risk in a typical industrial and mining gathering area was assessed. The integrated risk includes the risk level of the soil environment and inherent risk level of the polluting factories.

In industrial and mining gathering areas, the theories and methods of risk assessment and management of the regional soil environment remain at the developmental stage. In particular, the spatial and temporal zoning of environmental risk, multi-risk coupling and risk-field superimposition, and the allocation capacities of regional environmental risk are still being explored. Industrial and mining gathering areas have already implemented technologies and management systems to alleviate their integrated risk to the soil environment. However, further tests, optimization, and upgraded and improved application practices are needed, which should be based on the investigation and evaluation of risk sources.

## Appendix

### Appendix A. Assessment of Human Health Risk

Soil contaminants enter the human body mainly via the food chain, oral ingestion, skin contact, and breathing [12,29]. The health impacts of soil pollutants predominantly arise by the distribution and migration of soils, crops, and food. Therefore, in industrial and mining gathering areas, where soil environments are largely contaminated by heavy metals, the oral intake of such pollutants poses the most serious threat. Among the pollutants investigated in the current study, three heavy metals (Cr^6+^, Cd, and As) and seven organics (listed in Table A1) were identified as carcinogenic by the Integrated Risk Information System (IRIS) of US Environmental Protection Agency (EPA) [30] and the *Technical Guidelines for Risk Assessment of Contaminated Sites (HJ 25.3-2014)* [31] issued by China’s Ministry of Environmental Protection. The non-carcinogenic contaminants are ten heavy metals and eight organics (listed in Table A2).

The oral intakes of carcinogenic and non-carcinogenic pollutants in residential land are calculated by Equations (A1) and (A2), respectively:

(A1) $$CDI_{car}=\frac{\left(\frac{IR_{c}\times ED_{c}\times EF_{c}}{BW_{c}}+\frac{IR_{a}\times ED_{a}\times EF_{a}}{BW_{a}}\right)\times ABS_{o}}{AT_{ca}}\times CS\times CF$$

(A2) $$CDI_{ncr}=\frac{IR_{c}\times ED_{c}\times EF_{c}\times ABS_{o}}{BW_{c}\times AT_{ca}}\times CS\times CF$$

Similarly, the oral intakes of carcinogenic and non-carcinogenic pollutants in farmland and industrial land are calculated by Equations (A3) and (A4), respectively:

(A3) $$CDI_{cafi}=\frac{IR_{a}\times ED_{a}\times EF_{a}\times ABS_{o}}{BW_{a}\times AT_{ca}}\times CS\times CF$$

(A4) $$CDI_{ncfi}=\frac{IR_{a}\times ED_{a}\times EF_{a}\times ABS_{o}}{BW_{a}\times AT_{nc}}\times CS\times CF$$

In the above expressions, *CDI_car_* and *CDI_ncr_*, respectively, denote the oral intakes (mg/kg·d) of carcinogenic and non-carcinogenic pollutants from residential land, and *CDI_cafi_* and *CDI_ncfi_* are the corresponding intakes from farmland and industrial land, respectively. The subscripts *a* and *c* refer to adults and children, respectively. *IR_c_* and *IR_a_* denote the daily intake from soil (mg/d), and *ED_c_* and *ED_a_* are the periods of exposure duration (year) (the corresponding frequencies (d/year) are *EF_c_* and *EF_a_*). *BW_c_* and *BW_a_* denote average body weights (kg), *ABS_o_* is the oral intake absorption efficiency factor, and *AT_ca_* and *AT_nc_* are the average times of the carcinogenic and non-carcinogenic effects, respectively (d). *CS* is the pollutant content in the soil (mg/kg), and *CF* is a conversion factor.

Total carcinogenic and non-carcinogenic risks of various soil pollutants are calculated by formulas (A5) and (A6), respectively:

(A5) $$TCR=\sum_{i=1}^{n}CDI_{cai}\times SF_{oi}$$

(A6) $$THI=\sum_{i=1}^{n}\frac{CDI_{nci}}{RfD_{oi}}$$

In these formulas, *TCR* and *THI* denote the total carcinogenic and non-carcinogenic risks, respectively, *CDI_cai_* and *CDI_nci_* are the oral intakes (mg/kg·d) of a single pollutant and non-carcinogenic pollutant, respectively, *i* denotes an individual pollutant, *SF_oi_* is the carcinogenic slope factor of the *i*-th pollutant (kg·d/mg) (listed in Table A1), and *RfD_oi_* is the reference oral intake dose of the *i*-th pollutant (mg/kg·d) (listed in Table A2).

The parameter values in the above formulas are listed in Table A1, Table A2 and Table A3, the values were extracted from the *Technical Guidelines for Risk Assessment of Contaminated Sites (HJ 25.3-2014)* [31], issued by China’s Ministry of Environmental Protection.

**Table A1:** Carcinogenic slope factor (SF_o_) of carcinogenic pollutants (From the *Technical Guidelines for Risk Assessment of Contaminated Sites HJ 25.3-2014*).

Pollutant	SF_o_(kg·d/mg)
As	1.50
Cd	0.61
Cr^6+^	0.50
Benzene	5.50 × 10^−2^
Ethylbenzene	1.10 × 10^−2^
Carbon tetrachloride	7.00 × 10^−2^
Benzo (a) pyrene	7.30
Dibenzo (a, h) anthracene	7.30
Dichloroethane, 1,2-	9.10 × 10^−2^
Benzo (b) fluoranthene	7.30 × 10^−1^

**Table A2:** Reference oral intake dose (RfD_o_) of non-carcinogenic pollutants (From the *Technical Guidelines for Risk Assessment of Contaminated Sites HJ 25.3-2014*).

Pollutant	RfD_o_(mg/kg·d)	Pollutant	RfD_o_(mg/kg·d)
Ni	2.00 × 10^−2^	Pb	3.50 × 10^−3^
Zn	3.00 × 10^−1^	Pyrene	3.00 × 10^−2^
Hg	3.00 × 10^−4^	Carbon tetrachloride	4.00 × 10^−3^
Co	3.00 × 10^−4^	Dichloroethane, 1,2-	6.00 × 10^−3^
V	9.00 × 10^−3^	Trichlorothane, 1,1,1-	2.00
As	3.00 × 10^−4^	Benzene	4.00 × 10^−3^
Cd	1.00 × 10^−3^	Ethylbenzene	1.00 × 10^−1^
Cr^6+^	3.00 × 10^−3^	Fluoranthene	4.00 × 10^−2^
Cu	4.00 × 10^−2^	Xylenes	2.00 × 10^−1^

**Table A3:** Exposure assessment parameters (From the *Technical Guidelines for Risk Assessment of Contaminated Sites HJ 25.3-2014*).

Exposure Parameter	Unit	Farmland and Industrial Land	Residential Land	
			Children	Adults
IR	mg/d	100	200	100
BW	kg	55.9	15.9	55.9
CF	kg/mg	10^−6^	10^−6^	10^−6^
EF	d/a	250	350	350
ED	a	25	6	24
ABS	/	1	1	1
ATca	d	26,280	26,280	
ATnc	d	9125	2190	

In general, acceptable values of the total carcinogenic risk range from 1 × 10^−6^ to 1 × 10^−4^, and the total non-carcinogenic risk value (*THI*) should not exceed 1. Therefore, on the basis of the calculation of the total carcinogenic risk, 1 × 10^−5^ was selected as an acceptable level of carcinogenic risk in the present study. The adjusted valuation criteria for the *TCR* are as follows: *TCR* ≤ 1 × 10^−5^ (low risk), 1 × 10^−5^ < *TCR* ≤ 1 × 10^−4^ (moderate risk), 1 × 10^−4^ < *TCR* ≤ 5 × 10^−4^ (high risk), and *TCR* > 5 × 10^−4^ (extreme risk). The *THI* was divided into two risk levels: *THI* < 1 (no risk) and *THI* ≥ 1 (non-carcinogenic risk).

### Appendix B. Evaluation Index System and Weight Distribution

The polluting enterprises in industrial and mining gathering areas significantly differ in type, pollutant emission characteristics, and risk supervision level. Therefore, the environmental risks also differ among enterprises. To comprehensively assess the inherent risk level of polluting enterprises, this study applies an evaluation index system comprising sudden risk, cumulative risk, and risk supervision. Sudden risk refers to the risks on soil environment and human health posed by unexpected environmental accidents, natural disasters, and other transient factors. The sudden risk might reflect the degree to which various factors of polluting enterprises affect the environment. These factors include design layout, technology, management skills, and quality of personnel. Cumulative risk refers to the potential damage to human health and ecological environments by long-term, non-accidental discharge of pollutants during human production and development activities. The cumulative risk could reflect the risk imposed on soils during normal operation of polluting enterprises. Risk supervision focuses on the production safety, elimination of hazards, and treatment of contaminants. Risk supervision reflects the ability of the polluting enterprise to effectively control and prevent risks, and indirectly reflects the enterprises’ handling of production processing, equipment, management, and safety hazards.

To comprehensively assess the risk of polluting enterprises, each index must be weighted to reflect its contribution to the evaluated risk. Indices are commonly weighted by their entropy, Delphi method, the analytic hierarchy process (AHP), or principal component analysis (PCA). The present study adopts a mix of qualitative and quantitative methods. Advice was sought from 20 experts (including five environmental regulators, five engineers specializing in environmental impact assessment, and ten professors engaged in soil environmental protection). The indicator weights of the three evaluations in the criteria layer were then distributed by the AHP and Delphi methods on the basis of the characteristics of the soil environment and the indicators’ individual contributions to the soil quality in the area. The judgment matrix was constructed as **S** = (*u_ij_*)_m×n_ (A7):

(A7) $$\text{S}=\left|\begin{array}{ccc} {\text{u}}_{11} & \ldots & {\text{u}}_{1\text{m}}\\ \vdots & \ddots & \vdots \\ {\text{u}}_{\text{m}1} & \cdots & {\text{u}}_{\text{mm}} \end{array}\right|$$

where *u_ij_* denotes the importance of indicator *i* to indicator *j*.

The consistency of the judgment matrix can be tested through calculation. The consistency is acceptable when the consistency proportion < 0.1. The feature vector **A** = (w_1_,w_2_,…,w_n_) indicates that the largest eigenvalue of the judgment matrix corresponds to the weight distribution. The weight distribution results are presented in Table A3.

### Appendix C. Standardized Indicators and Calculation Method

**Table A4:** Grading standards of the assessment indicators.

Indicators	Classification Standards			
	0	1	2	3
Inventory level of hazardous substances	<1	[1, 10)	[10, 100)	≥100
Service life of equipment	≤5	(5, 10]	(10, 20]	>20
Environmental emergency response plan	Developed, accredited, and filed	Developed and accredited	Developed	Not developed
Emergency rescue personnel	Professional emergency personnel	/	Part-time emergency personnel	No
Environmental emergency drills frequency	At least once a year, with records and reports	At least once a year, without records and reports	/	No
Number of environmental emergencies in last three years	No	1	2	4
Industrial policy requirements	Encourage	/	Restriction	Eliminate
Construction Period (a)	≤5	(5, 10]	(10, 15]	>15
Industrial output value (10^4^ Yuan)	≥10^7^	[10^6^, 10^7^)	[10^5^, 10^6^)	<10^5^
Annual production time (h)	≤2000	(2000, 5000]	(5000, 7500]	> 7500
Annual emissions of soot (t)	≤0.1	(0.1, 1.5]	(1.5, 5]	>5
Annual emissions of sulfur dioxide (t)	≤10	(10, 100]	(100, 1000]	>1000
Annual emissions of nitrogen oxide (t)	≤0.1	(0.1, 1]	(1, 5]	>5
Industrial water recycling rate (%)	≥90	[70, 90)	[50, 70)	<50
Utilization rate of industrial solid waste (%)	≥90	[70, 90)	[50, 70)	<50
Online sewage monitoring system	Complete	/	Less monitoring project	No
Routine environmental monitoring capacity	Complete	Only water	Only air	No
Rain and sewage system	Established	/	/	No
Ground seepage treatment	Meet the requirements of seepage	/	/	No
Treatment rate of soot (%)	100	[80, 100]	[55, 80]	≤55
Treatment rate of sulfur dioxide (%)	100	[80, 100]	[55, 80]	≤55
Treatment rate of nitrogen oxide (%)	100	[80, 100]	[80, 100]	≤55

In Table A4, the inventory level *Q* of hazardous substances is determined by Equation (A8):

(A8) $$Q=\sum_{i=1}^{n}\frac{q_{i}}{Q_{i}}$$

where *Q_i_* and *qi* denote the inventory and critical value, respectively, of hazardous substance *i*, and *n* is the number of different hazardous substances. The critical values of hazardous substances are listed in *Enterprise Environmental Risk Level Assessment Method* [32]. Industrial output value refers to the total value of the products of the industrial enterprises within a certain period. Industrial policy requirements are accessible through the *Industrial Restructuring Catalog* issued by the National Development and Reform Commission [33]. Soot denotes the smoke and dust generated by industrial production process that performed in industrial furnaces using gas, liquid and solid fuels. According to the *National Industrial Soot Emission Standards* (GB T9078-1996), soot is an important indicator to measure the pollutant emission level of industrial factories in China. Indicator values were acquired mainly from pollution census, completion acceptance reports of enterprises, statistical yearbooks, and departmental rules and regulations from China’s State Council.

The comprehensive risk levels of polluting enterprises in mining and industrial gathering areas were characterized by comprehensive risk indexing (*CRI*) on the basis of the weight distributions and classification standards. The *CRI* is calculated by Equation (A9):

(A9) $$CRI=\sum_{i=1}^{n}w_{i}\times I_{i}$$

where *CRI* represents the inherent risk level of an enterprise, and *w_i_* and *I_i_* represent the weight and score of indicator *i*, respectively. The *CRI* lies within [0, 3] and is divided into four ranks; a score of [0, 1] indicates a healthy state of all indicators and a low risk level, (1, 2] denotes an alert state with moderate risk level, (2, 2.5] indicates a poor state with high risk level, and (2.5, 3] suggests extreme risk to human and environmental health.
